# Analysis of treatment planning time and optimization parameters for inverse planning for intracavitary and interstitial brachytherapy in uterine cervical cancer

**DOI:** 10.1002/acm2.70157

**Published:** 2025-07-14

**Authors:** Jun Tomihara, Jun Takatsu, Naoya Murakami, Noriyuki Okonogi, Tatsuya Inoue, Kotaro Iijima, Kotaro Matsuda, Naoto Shikama

**Affiliations:** ^1^ Department of Radiation Oncology Graduate School of Medicine Juntendo University Bunkyo‐ku Tokyo Japan; ^2^ Graduate School of Medicine Nagoya University Nagoya Aichi Japan; ^3^ Department of Radiology Juntendo University Urayasu Hospital Urayasu Chiba Japan

**Keywords:** brachytherapy, cervical cancer, HIPO, IC/ISBT, inverse planning, optimization

## Abstract

**Purpose:**

This study aimed to investigate the effect of inverse planning parameters on dose‐volume indices in brachytherapy for uterine cervical cancer.

**Methods:**

Fourteen consecutive patients with cervical cancer who received intracavitary and interstitial brachytherapy (IC/ISBT) were selected. Tandem, ovoid, and interstitial needles were used in all cases. The evaluation plans were recalculated from the first fraction of clinical brachytherapy plans. The correlation between the 11 dose optimization parameters of inverse planning and the 13 dose‐volume indices was evaluated. The parameters were adjusted in five levels, and dose optimization was performed in hybrid inverse planning optimization (HIPO). Spearman's rank correlation and multiple regression analyses were conducted to assess the association between the parameters and the indices. The indices included clinical target volume (CTV) dose, organ‐at‐risk (OAR) dose, homogeneity, and conformity. Additionally, the correlation between optimization parameters and calculation time was investigated, along with a technique for efficiently generating treatment plans.

**Results:**

“CTV Max Weight” and “OAR Max Weight” were the key parameters significantly affecting the indices. Increasing “CTV Max Weight” improved homogeneity but reduced the target coverage. The effect of “OAR Max Weight” on the dose reduction of CTV_HR_ D_90_ (β = −0.59) was more significant than that on the dose reduction of OAR D_2cc_ (β = −0.21). In addition, adjusting “CTV Min Weight” and “CTV Volume” could reduce the hyper‐dose sleeve without increasing the OAR dose. A large number of normal tissue sampling points could negatively affect the dose distributions and increase the calculation times.

**Conclusion:**

“CTV Max Weight” and “OAR Max Weight” were the most influential parameters in HIPO, significantly affecting dose‐volume indices in IC/ISBT for uterine cervical cancer. Additionally, parameters that regulate the hyper‐dose sleeve and needle‐delivered dose were identified. The quality of treatment planning can be maintained and planning time reduced by appropriately optimizing these parameters.

## 1 INTRODUCTION

Combined external beam radiation therapy (EBRT) and brachytherapy (BT) is the standard treatment for cervical cancer.^1^, ^2^ In recent years, the additional use of interstitial needles in BT has enabled adequate dose delivery to irregularly shaped and/or bulky tumors. The role of BT in cervical cancer treatment has become increasingly important.^3^ A major challenge in the clinical introduction of intracavitary and interstitial BT (IC/ISBT) is the increased planning time, which results from the insertion of interstitial needles into the tumor and the complexity of dose optimization. Takagawa et al. reported that compared to ICBT, IC/ISBT significantly increased the time required not only for application insertion but also for treatment planning.^4^ The difference between IC/ISBT and EBRT is that high‐quality treatment plans are required to be generated within a limited time for the former. The quality of the treatment plan in IC/ISBT could be strongly influenced by the skill of the planner, and the treatment planning time would vary considerably between planners due to the high degree of freedom in treatment planning. Extending the treatment planning time could result in a burden on both the patient and the medical staff.

It has been 20 years since inverse planning became possible in treatment planning for IC/ISBT, as well as EBRT.^5^ The implementation of inverse planning in IC/ISBT can ensure the quality of treatment plans and reduce treatment planning time. Planning studies for inverse planning in uterine cervical cancer,^6^, ^7^, ^8^, ^9^ prostate cancer,^10^, ^11^ breast cancer,^12^ and head and neck cancer^13^, ^14^ have been reported. However, its use in clinical practice remains limited. Previous studies have mainly compared manual planning with inverse planning and the different algorithms used in inverse planning.^6^, ^7^, ^9^, ^10^, ^13^, ^14^, ^15^ Similar to EBRT, the ideal parameter configurations for inverse planning could differ per case due to anatomical variations. Thus, an understanding on how to adjust the optimization parameters is essential for the clinical implementation of inverse planning.^12^, ^16^ A method that can decrease the hyper‐dose sleeve, which exceeds 200% of the prescribed dose (PD), specifically in BT using inverse planning, has not been established. In addition, a technique for limiting the dose contribution of additional needles is yet to be developed.

This study aimed to validate the association between various optimization parameters of inverse planning and dose‐volume indices in IC/ISBT for cervical cancer.

## 2 MATERIALS AND METHODS

This study was conducted on 14 consecutive patients with cervical cancer who received curative IC/ISBT between January 2023 and October 2023. EBRT was delivered in 25–28 fractions at 45–50 Gy without central shielding, and CT‐based image‐guided adaptive BT was delivered in 3–4 fractions. The doses delivered during BT were converted to the biological equivalent dose in 2 Gy per fraction (EQD_2_). The minimum dose delivered to 90% of the high‐risk clinical target volume (CTV_HR_) D_90_ was calculated using EQD_2_. The total dose aimed to exceed 85 Gy, CTV_HR_ D_90_.^1^ Fractional dose constraints vary depending on the number of IC/ISBT fractions.^17^ All patients in this study were treated according to these protocols. The institutional clinical review board of our institution (E23‐0098) approved this study. The median age of the patients was 52.5 (range: 30–72) years, and eight patients presented with T2 disease and six with T3 disease. Tandem, ovoid, and interstitial needles were used in all cases. For tandem and ovoid applicators, either the Fletcher‐style or Geneva (Elekta AB, Stockholm, Sweden) applicators were used. Additional interstitial needles with a median number of 2.0 (range: 1–7) were inserted. To spare the dose in rectum and bladder,^17^, ^18^, ^19^ hyaluronic acid gel (MucoUp, Seikagaku Co., Tokyo, Japan) of 5–30 mL was injected as a spacer into the rectovaginal and vesicouterine septum.

Treatment planning CT images were acquired in 2‐mm slices using Aquilion LB (Cannon Medical Systems, Otawara, Japan) in the treatment room. BT was performed using ^192^Ir microSelectron HDR remote afterloader (Elekta AB) with Oncentra version 4.6.3 (Elekta AB) as the treatment planning system. The calculation grid was set at 1 mm. The ^192^Ir sources were placed in the applicators at 5‐mm steps. The CTV_HR_ was delineated according to the recommendations of the Japanese Radiation Oncology Study Group.^20^ The organs‐at‐risk (OARs) for the bladder, rectum, sigmoid colon, and small bowel were contoured by an experienced radiation oncologist. The median CTV_HR_ volume was 34.7 cc (range: 16.3–163.5 cc).

Oncentra implements two inverse planning algorithms: inverse planning simulated annealing (IPSA) and hybrid inverse planning optimization (HIPO). Previous studies have compared IPSA and HIPO, and results showed no significant differences between the two algorithms.^7^, ^9^, ^14^, ^21^ This study utilized HIPO because of its user‐friendly optimization approach that allows users to select the applicator needle for inverse planning while locking the tandem and ovoid dwell time in manually set numbers.^7^, ^13^, ^22^

Table 1 shows the initial dose optimization parameters used in HIPO. The optimization parameters are described below. Further details on the functions of these optimization parameters can be found in the Elekta White Paper.^22^

**TABLE 1:** Initial setting values of the optimization parameters used in HIPO plans.

	Min weight	Max weight	Min value (cGy)	Max value (cGy)	Sampling points	Volume	Surface	% on Surface	Density	DTGR
Bladder		1		550	1000	500	500	50		0.5
CTV_HR_	100	1	600	1200	1149	100	1049		10	
Rectum		1		500	1000	500	500	50		
Sigmoid		1		550	1000	500	500	50		
Small bowel		1		550	1000	500	500	50		
Normal tissue		1		400	10 000					

**Max/Min weight**: The strength of the dose constraints in the optimization calculation.

**Max/Min value**: Dose constraint.

**Sampling points**: Setting the number of samplings for the surface and inside of each structure.

**Volume**: Number of sampling points within the region of interest (ROI).

**Surface**: Number of sampling points on the ROI surface.

**% on surface**: Percentage of surface sampling points.

**Density**: Density of sampling points on the CTV_HR_ surface.

**Dwell time gradient restriction (DTGR)**: Limits the variation in dwell time between adjacent dwell positions. The higher the DTGR value, the smaller the variation in dwell time between adjacent dwell positions. It can be set in the range of 0.0–1.0.

The sampling points, representing the number of dose calculation points, can be manually adjusted within the region of interest such as “CTV Volume” and “CTV Density.” Among them, DTGR plays a key role in regulating variations in dwell times between adjacent dwell positions.

Based on the settings presented in Table 1, the range of optimization parameters used in this study are presented in Table 2. When adjusting individual parameters, all other parameters remain fixed at their initial values. The 630 HIPO plans were generated by adjusting each parameter across five levels. The optimization parameters in CTV_HR_ and OARs were modified individually. “Max Value” of OARs were also adjusted individually from the initial setting values. These plans were evaluated with 13 dose‐volume indices based on the recommendations of GEC ESTRO GYN.^23^ The dose‐volume indices analyzed included CTV_HR_ D_90_, D_98_, the volume of CTV_HR_ receiving 100% of the PD (V_CTV,100_), and the volumes receiving 150% and 200% of the PD (V_150_, V_200_). The OARs for D_2cc_ in the bladder, rectum, sigmoid colon, and small bowel were evaluated. In addition, the dwell time for each applicator and needle was evaluated. The coefficient of variation (CV) for each applicator was used to evaluate the variation in the dwell time at each dwell position. The CV was calculated by dividing the standard deviation of the dwell time for each applicator by the average value. The conformity index (CI) was calculated to assess the PD coverage of the target volume and exclude nontarget volumes.^24^ The equation is as follows:

(1) $$\mathrm{CI}={V^{2}}_{\mathrm{CTV},100}/\left(V_{\mathrm{CTV}}\times V_{100}\right)$$

**TABLE 2:** Range of setting values for the applied optimization parameters.

Parameters	Targets	Values
Min weight	CTV_HR_	20, 40, 60, 80, 100
Max weight	CTV_HR_	1, 3, 10, 30, 100
	OAR	1, 3, 10, 30, 100
Max value (cGy)	CTV_HR_	600, 900, 1200, 1500, 1800
	OAR	−200, −100, ±0, +100, +200
Volume	CTV_HR_	30, 100, 300, 1000, 3000
Total sampling points	OAR	100, 300, 1000, 3000, 10 000
	Normal tissue	300, 1000, 3000, 10 000, 30 000
Density	CTV_HR_	1, 3, 10, 30, 100
% on Surface	OAR	20, 35, 50, 65, 80
DTGR		0.1, 0.3, 0.5, 0.7, 0.9

The homogeneity index (HI) was used to measure the uniformity of the dose distribution within the CTV_HR_.^10^ The equation is as follows:

(2) $$\mathrm{HI}=\left(V_{100}-V_{150}\right)/V_{100}$$

The dose nonuniformity index (DNR) was calculated from 1.5 times the PD, and it indicates the percentage of high doses.^25^ The equation is as follows:

(3) $$\mathrm{DNR}=V_{150}/V_{\mathrm{CTV}}$$

Initially, a Spearman's rank correlation analysis was performed to examine the correlations between the 11 parameters and the 13 dose‐volume indices. This analysis identified direct relationships between specific optimization parameters and dose‐volume indices. In particular, the aim was to clarify the independent effects of parameter variations on individual dose‐volume indices. This approach allowed planners to efficiently identify key parameters that should be adjusted to achieve specific clinical objectives, such as improving target coverage or reducing OAR doses. The correlation coefficient (*r*) indicated the strength of the correlation with an absolute value ≧ 0.7, which suggested a high correlation.

Next, a multiple regression analysis was conducted to determine which parameters had a significant effect on each index. The regression equations were derived by adding the 11 parameters together, with significant parameters identified via this analysis. In clinical practice, the dose distribution is determined by the complex interaction of multiple parameters, not by a single parameter. This analysis quantified the relative impact of each parameter on specific dose‐volume indices using standardized regression coefficients (β). The β represents the degree of influence, with larger values indicating a stronger influence. Variance inflation factors (VIFs) were used to evaluate multicollinearity in the regression analysis. This approach enabled planners to identify the most effective combination of parameter settings and to develop strategies for achieving optimal dose distributions with increased efficiency. R ver. 4.2.2 (R Foundation, Vienna, Austria) was used. A *p* value < 0.05 indicated statistically significant differences.

To demonstrate that HIPO can be applied to irregularly shaped tumors, ellipticity was measured additionally. The ellipticity was defined as a ratio of the maximum and minimum diameters in the axial slice where the CTV_HR_ cross‐sectional area was greatest.

ChatGPT was utilized to enhance the readability of this publication. The authors comprehensively reviewed and edited the content following its use and took full responsibility for the final version of the publication.

## 3 RESULTS

**FIGURE 1:**
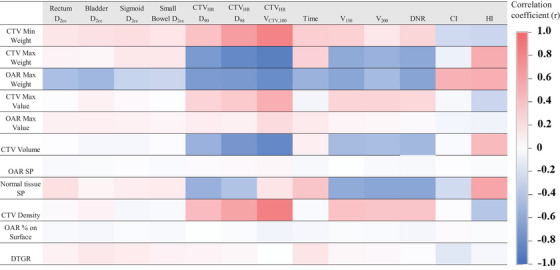
Association between the optimization parameters and the dose‐volume indices based on the Spearman's rank correlation analysis. The colors represent the different values of the correlation coefficient (*r*): red indicates a positive correlation (0 < *r* < 1); blue, a negative correlation (−1 < *r* < 0); and white, no correlation (*r* = 0).

**FIGURE 2:**
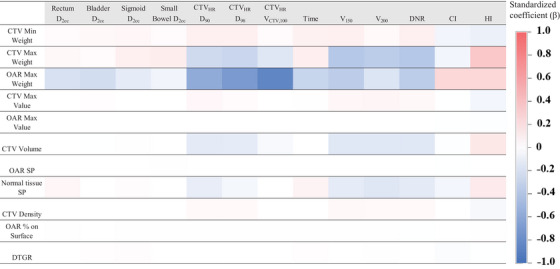
Effect of each optimization parameter on the dose‐volume indices based on the multiple regression analysis. The colors represent the different values of the standardized regression coefficient (β): red indicates a positive effect (0 < β < 1); blue, a negative effect (−1 < β < 0); and white, no effect (β = 0).

The correlation between each parameter and the dose‐volume indices and the impact of each parameter on the dose‐volume indices are shown in Figures 1 and 2. The values of r and β are provided in the supplemental materials (Table S1 and S2).

The results of Spearman's rank correlation analysis presented in Figure 1 show that “CTV Max Weight” and “OAR Max Weight” were significantly negatively correlated with CTV_HR_ D_90_, D_98_, and V_CTV,100_. In particular, “CTV Max Weight” was negatively correlated with CTV_HR_ D_90_ (*r* = −0.70, *p* < 0.01) and V_CTV,100_ (*r* = −0.90, *p* < 0.01). Meanwhile, “OAR Max Weight” had similar negative correlations with CTV_HR_ D_90_ (*r* = −0.69, *p* < 0.01) and V_CTV,100_ (*r* = −0.80, *p* < 0.01). In addition, “CTV Min Weight,” “CTV Volume,” and “CTV Density” had strong correlations with CTV_HR_ V_CTV,100_ (|*r*| > 0.80).

The results of multiple regression analysis exhibit the degree of parameter influence (Figure 2), independent of the strength of the correlation (Figure 1). Figure 2 shows that “CTV Max Weight” and “OAR Max Weight” significantly affected almost all dose‐volume indices. Reducing “CTV Max Weight” led to increases in CTV_HR_ D_90_ and V_ctv,100_, but not in OAR D_2cc_. Moreover, the effect of “OAR Max Weight” on CTV_HR_ D_90_ (β = −0.59, *p* < 0.01) was greater than that on OAR D_2cc_ (β = −0.21 to −0.08, *p* < 0.05). In addition, decreasing “CTV Min Weight” reduced the high‐dose indices, such as DNR and V_150_. Similarly, increasing “CTV Volume” led to reductions in DNR, V_150_, and V_200_. All VIFs were < 2, which indicated the absence of significant multicollinearity among the indices.

**FIGURE 3:**
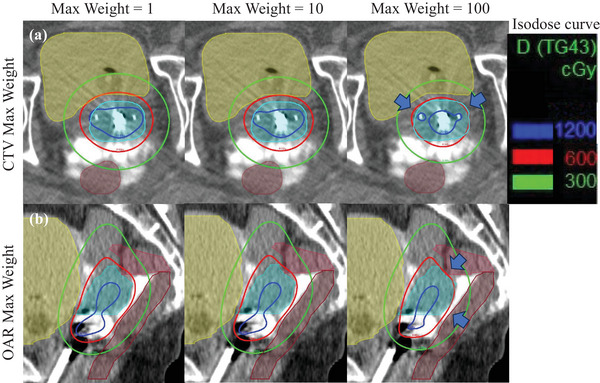
Example of the effect of (a) “CTV Max Weight” and (b) “OAR Max Weight” on the dose distributions. The green, red, and blue lines represent the isodose curves of 300, 600, and 1200 cGy, respectively. The region of light blue is CTV_HR_. The brown, yellow, and red regions represent the rectum, bladder, and sigmoid colon, respectively. The arrows indicate where the 600 cGy isodose curve shrinks.

Figure 3 shows an example of the effect of “CTV Max Weight” and “OAR Max Weight” on dose distributions. Table 3 shows the ratio of needle dwell time for each applicator according to the optimization parameters. The needle dwell time ratios represent the mean values of individual cases.

**TABLE 3:** The dwell time and ratio of each applicator during “CTV Max Weight” and “OAR Max Weight” changes.

	Dwell times		
CTV max weight	1	10	100
Tandem (s)	149.3	158.0	219.1
Ovoid (s)	111.0	158.6	163.8
Needle (s)	125.8	114.5	110.7
N/T&O ratio	0.58	0.45	0.38

OAR max weight	1	10	100
Tandem (s)	149.3	152.7	174.8
Ovoid (s)	111.0	106.0	74.7
Needle (s)	125.8	119.8	117.6
N/T&O ratio	0.58	0.52	0.52

Abbreviation: N/T&O ratio, dwell time ratio of needles to tandem and ovoid.

Table 3 shows that as “CTV Max Weight” increased, the dose delivered to the CTV_HR_ from the tandem and ovoid increased. Meanwhile, the dose delivery from the needles decreased. In Figure 3a, as “CTV Max Weight” increased, the volume surrounded by the 6 Gy isodose line decreased. If “CTV Max Weight” was set from 1 to 100, the median V_CTV,100_, V_150_, and V_200_ values decreased from 99.9% to 96.9%, from 73.6% to 51.5%, and from 42.6% to 20.7%, respectively. Table 3 also depicts that increasing “OAR Max Weight” increased the dwell time of the tandem but decreased that of the ovoid. The ratio of needles to tandem and ovoid for the different “OAR Max Weight” were almost similar. In Figure 3b, increasing “OAR Max Weight” values caused the 6 Gy isodose curve to shift slightly away from the rectum and sigmoid colon to reduce the dose to the OARs. If “OAR Max Weight” was set from 1 to 100, the median D_2cc_ values for the rectum, bladder, sigmoid, and small bowel decreased from 388.5 to 211.5 cGy, from 553.0 to 345.0 cGy, from 346.0 to 207.0 cGy, and from 179.0 to 116.0 cGy, respectively. Similarly, the median CTV_HR_ D_90_, V_150_, and V_200_ values reduced from 756 to 522.5 cGy, from 73.6% to 46.0%, and from 42.6% to 28.2%, respectively.

**FIGURE 4:**
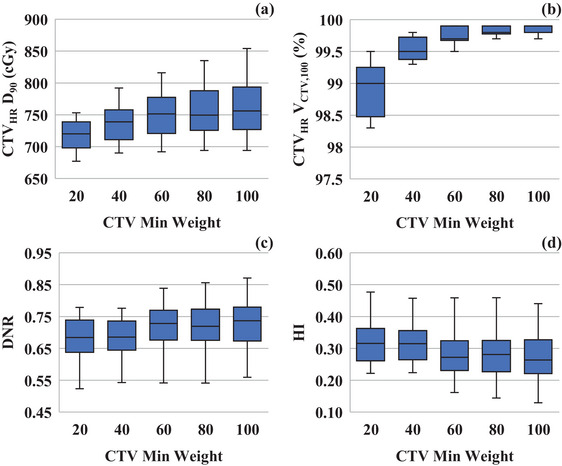
Effect of “CTV Min Weight” on the dose‐volume indices. Association between “CTV Min Weight” and (a) CTV_HR_ D_90_; (b) CTV_HR_ V_CTV,100_; (c) Dose nonuniformity index; (d) Homogeneity index.

As presented in Figure 4a, increasing “CTV Min Weight” significantly increased CTV_HR_ D_90_. As “CTV Min Weight” increased from 1 to 10 and 100, the median CTV_HR_ V_CTV,100_ improved from 99.0% to 99.7% and 99.9%, respectively (Figure 4b). In addition, as the CTV_HR_ dose increased, the high‐dose region expanded, causing DNR to increase and HI to worsen (Figure 4c,d).

**FIGURE 5:**
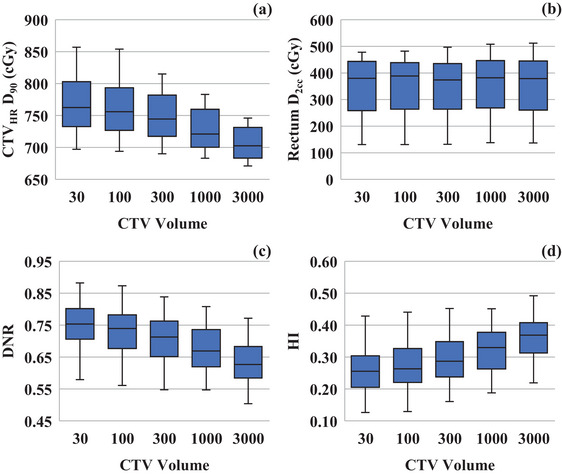
Effect of “CTV Volume” on the dose‐volume indices. Association between **“**CTV Min Weight” and (a) CTV_HR_ D_90_; (b) rectum D_2cc_; (c) Dose nonuniformity index; (d) Homogeneity index.

Increasing “CTV Volume” reduced the CTV_HR_ dose, without significant differences in the rectum D_2cc_ (Figure 5a,b). In addition, the high‐dose region shrunk, resulting in a reduction in DNR and an improvement in HI (Figure 5c,d).

**FIGURE 6:**
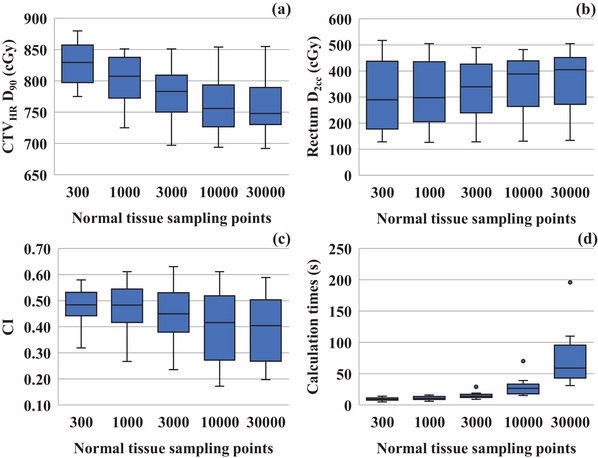
Effect of the normal tissue sampling points on the dose‐volume indices. Association between “Normal tissue sampling points” and (a) CTV_HR_ D_90_; (b) rectum D_2cc_; (c) conformity index; (d) calculation time by HIPO. Circles represent the longest calculation time for the largest tumor volume (163.5 cc).

As shown in Figure 6, as normal tissue sampling points increased from 300 to 30 000, the median values of CTV_HR_ D_90_, rectum D_2cc_, and CI were changed from 829.5 to 748.0 cGy, from 289.5 to 405.0 cGy, and from 0.48 to 0.40, respectively. The median calculation times were 9.0, 14.0, and 59.0 s for the sampling points of 300, 3000, and 30 000, respectively.

In Table 4, the CV in dwell time within individual applicators decreased as DTGR changed from 0.1 to 0.9. The mean values decreased from 0.60 to 0.08, from 0.71 to 0.17, and from 0.60 to 0.21 for the ovoid, tandem, and needle, respectively.

**TABLE 4:** The coefficient of variation of dwell time for each applicator.

	DTGR	0.1	0.3	0.5	0.7	0.9
Coefficient of variation	Ovoid	0.60	0.23	0.14	0.10	0.08
	Tandem	0.71	0.40	0.29	0.25	0.17
	Needle	0.60	0.42	0.31	0.26	0.21

Abbreviation: DTGR, dwell time gradient restriction.

The results of investigation into the tumor's irregular shape using the ellipticity ratio were the median of 2.50 (range: 1.81–4.52). With the initial HIPO parameter settings shown in Table 1, the median of V_CTV,100_ was 99.9% (range: 99.7–99.9%) and the median of CTV_HR_ D_90_ was 756 cGy (range: 694–854 cGy).

## 4 DISCUSSION

As shown in Figure 2, the most effective parameters were “CTV Max Weight” and “OAR Max Weight” due to their influence on nearly all indices. When using HIPO, these two parameters should be prioritized for adjustment to achieve appropriate dose‐volume indices. Proper modification of these parameters could generate the optimal dose distribution.

Increasing “CTV Max Weight” redistributed the dose delivery within the CTV_HR_, improving HI but reducing target coverage. As shown in Figure 3a, increasing “CTV Max Weight” would result in insufficient dose delivery from the needle, especially for irregular‐shaped tumors. Figure 2 also shows that “CTV Max Weight” has a greater impact on V_150_ and V_200_ than D_90_ and V_CTV,100_. The dwell time of the tandem and ovoid increased as shown in Table 3, which would typically increase high‐dose regions. However, increasing “CTV Max Weight” redistributed the CTV_HR_ dose, leading to an improvement in HI. Consequently, the high‐dose regions were reduced, resulting in decreases in V_150_ and V_200_. Trnková et al. found that the implementation of HIPO enabled the restriction of the hyper‐dose sleeve,^8^ which was consistent with our results. Based on this finding, adjusting “CTV Max Weight” is crucial for achieving a uniform dose distribution and suppressing high‐dose regions. Therefore, a lower “CTV Max Weight” should be selected to achieve a high target coverage. By contrast, when a higher “CTV Max Weight” is used to reduce the hyper‐dose sleeve, manual adjustments are required to determine whether or not to allow dose delivery to the underdose region.

Notably, “OAR Max Weight” not only reduces OAR dose but also affects CTV dose. Major et al. investigated the effects of “Max/Min Weight” and “Max/Min Value” parameters on the target via dosimetry.^12^ Further, this study performed the first detailed analysis of various HIPO parameters, categorizing them into CTV and OAR. “OAR Max Weight” is a parameter designed to reduce the OAR dose. However, “OAR Max Weight” had a greater effect on reducing the CTV_HR_ D_90_ compared with the OAR D_2cc_. Table 3 also shows that increasing “OAR Max Weight” decreased the dwell time of the ovoid, particularly as it was generally near the rectum than the tandem. The ratio of dose delivery from the tandem to the CTV_HR_ increased. However, because the overall CTV_HR_ D_90_ reduced, the V_150_ and V_200_ also decreased. At this time, locking the dwell time of the ovoid to ensure the CTV_HR_ dose could be a viable option. Therefore, when adjusting the OAR dose using “OAR Max Weight,” the CTV_HR_ dose must also be considered.

“CTV Min Weight” and “CTV Volume” could be the next most important dose optimization parameters. In Figure 4b and c, lowering “CTV Min Weight” reduced DNR. However, it had a weak impact on CTV_HR_ V_CTV,100_. Therefore, “CTV Min Weight” could be used to reduce the hyper‐dose sleeve while ensuring dose coverage. A trade‐off exists between “CTV Max Weight” and “CTV Min Weight” regarding dose conformity and homogeneity.^12^ Lowering “CTV Max Weight” ensures dose coverage. Nevertheless, it could increase the hyper‐dose sleeve. In such cases, reducing “CTV Min Weight” can suppress these high doses without significantly reducing the dose coverage. Therefore, when “CTV Max Weight” is set low, adjusting “CTV Min Weight” downward can help suppress the hyper‐dose sleeve effectively.

Similarly, “CTV Volume” could also be used to adjust the hyper‐dose sleeve. Figure 5a and b show that “CTV Volume” primarily affects the CTV_HR_ dose, without significantly changing the OAR dose. In particular, for head and neck cancer BT, an optimal dose distribution features a high HI with a minimal hyper‐dose sleeve. Therefore, to reduce the hyper‐dose sleeve while maintaining a dose distribution with a high HI, “CTV Volume” should be increased during adjustments.

Extremely large normal tissue sampling points often have a negative impact. An inappropriate number of sampling points could reduce the quality of treatment planning.^6^ If the number of normal tissue sampling points is remarkably large, the effect of suppressing the dose outside the CTV will be excessive, even reducing the dose delivered to the CTV itself. As shown in Figure 6a, increasing the sampling points led to a decrease in CTV_HR_ D_90_. As a result, the CI worsened, causing dose leakage into the OAR and OAR D_2cc_ growth. As shown in Figure 6d, the case with the largest tumor volume (163.5 cc) also had the longest calculation time (196 s at SP = 30,000). Spearman's correlation analysis between tumor volume and calculation time revealed a strong positive correlation (*r* = 0.84, *p* < 0.01). Calculation time increased with tumor size in inverse planning. Moreover, a larger number of sampling points increases the calculation time. For example, a dose optimization performed with 10 000 sampling points took a median of 26.5 s. Meanwhile, with 30 000 sampling points, it took 59.0 s—which is more than double the calculation time. Therefore, to ensure both quality and efficiency in treatment planning, the number of sampling points should be kept as low as possible, ideally below 10 000.

Menon et al. reported that the median time required for catheter reconstruction and dose optimization was 1 h (range: 0.3–2 h).^26^ Similarly, Takagawa et al. found that the median time for dose optimization in IC/ISBT procedures was 61.6 min (range: 40–102 min).^4^ In contrast, the present study showed that dose calculation using HIPO required a median of only 26.5 s for 10 000 SPs. In clinical practice, treatment planning is rarely completed in a single optimization and typically involves multiple adjustments. In this study, each optimization required less than one minute, and the results clarified which optimization parameters should be adjusted to achieve an optimal dose distribution. Compared with previous studies, these findings indicated that HIPO could substantially reduce planning time.

As shown in Figure 2, no significant influence was observed between DTGR and the dose‐volume indices. However, this does not imply that DTGR was not a significant optimization parameter. Discontinuous variations in dwell time at neighboring steps could lead to under‐ or overdosage distribution.^7^, ^13^ Table 4 shows the CV of the dwell time at adjacent dwell positions when DTGR varied. Within the same applicator, it was reconfirmed that DTGR forms a smooth dose distribution as it increases. Roy et al. investigated the dwell time deviation constraint (DTDC), an IPSA parameter that restricts deviations in dwell time, similar to DTGR in HIPO.^16^ As DTDC increased, HI improved, and V_200_ decreased. However, the results of this work did not confirm a clear association between DTGR and HI or V_200_ (Figures 1 and 2). The DTGR could reduce individual hot spots by smoothing the dwell time distribution. However, its effect could not be significant enough to produce a clear trend across the whole dose distribution. Balvert et al. also reported that limiting dwell time modulation did not improve the plan quality for prostate high‐dose rate BT.^11^ Therefore, DTGR allows for adjustments in the dwell time distribution. However, it does not necessarily affect the quality of the plan as evaluated by the dose‐volume indices. According to the recommendations in the Elekta White Paper,^22^ a value of approximately 0.5 is appropriate for DTGR.

Okonogi et al. evaluated the ellipticity on MR axial images and used it as a guide for needle insertion when the ellipticity was 2 or greater.^27^ In this study, the ellipticity was evaluated from the planning CT images in which the applicator and needle had already been inserted. The median ellipticity of the CTV_HR_ in the cases selected for this study was 2.50 (range: 1.81–4.52). Assuming that the tandem was inserted into the center of the CTV_HR_, the findings of this study suggest that HIPO could be used to generate dose distributions for a wide range of irregularly shaped tumors. It was also concluded from Figure 4a and b that HIPO could generate dose distributions that fit irregularly shaped tumors. It was also shown that the CTV_HR_ coverage could be adjusted for irregularly shaped tumors using “CTV Min Weight.” However, the limitation of this study was that the definition of ellipticity was based on the assumption that the tandem was inserted into the center of the CTV_HR_. However, the risk of the tandem being positioned outside the center of the large CTV_HR_ is limited in general intracavitary BT. When this happens, it should be corrected when the planning CT is scanned. Therefore, the definition of ellipticity in this study should not change the findings of this study. In future work, the number of cases will be increased further, and the correlation with the ellipticity in MR images will also be investigated.

The current study had several limitations. It was conducted utilizing CT images with the hydrogel spacer. In cases where in a hydrogel spacer was not used, there could have been a stronger correlation between “CTV Min Weight” and the D_2cc_ of OARs. However, the influence of the optimization parameters on the dose distribution did not change regardless of the presence of the hydrogel spacer. This study only included cases of IC/ISBT using a limited applicator type with the tandem/ovoid. Therefore, future studies should evaluate the optimization parameters for various applicators such as tandem/ring and various irradiation sites.

## 5 CONCLUSION

Using HIPO, key optimization parameters such as “CTV Max Weight” and “OAR Max Weight” in IC/ISBT were found to significantly influence the dose‐volume indices on dose optimization for uterine cervical cancer. In addition, we identified the parameters that suppress the hyper‐dose sleeve and those that regulate the dose delivered by needles. A proper adjustment of these parameters could improve planning efficiency while maintaining the quality of treatment plans. Future studies should evaluate the application of optimization parameters across different applicators and irradiation sites.

## AUTHOR CONTRIBUTIONS

Jun Tomihara collected and analyzed the data. Jun Tomihara and Jun Takatsu prepared the manuscript. Jun Takatsu is the corresponding author. All co‐authors revised the manuscript. Dr. Murakami and Dr. Shikama provided supervision of the manuscript. Dr. Shikama provided final approval of the manuscript.

## CONFLICT OF INTEREST STATEMENT

The authors declare no conflicts of interest.

## Supporting information



Supporting Table S1

Supporting Table S2
