# Supplementary material for: Analysis of treatment planning time and optimization parameters for inverse planning for intracavitary and interstitial brachytherapy in uterine cervical cancer
Source: J Appl Clin Med Phys. 2025 Jul 14;26(7):e70157. doi: 10.1002/acm2.70157 (PMC12257335; doi:10.1002/acm2.70157)
Supplement: Supplementary file 1 — Supporting Table S1 [file ACM2-26-e70157-s001.docx]

Table S1. Association between the optimization parameters and the dose-volume indices (Spearman’s rank correlation analysis).

|  |  | Rectum 　D_2cc_ | Bladder 　D_2cc_ | Sigmoid 　D_2cc_ | Small 　Bowel D_2cc_ | CTV_HR_ 　D_90_ | CTV_HR_ 　D_98_ | CTV_HR_ V_CTV,100_ | Time | V_150_ | V_200_ | DNR | CI | HI |
| --- | --- | --- | --- | --- | --- | --- | --- | --- | --- | --- | --- | --- | --- | --- |
| CTV Min Weight | r | 0.16 | 0.19 | 0.21 | 0.16 | 0.40 | 0.64 | **0.82** | 0.31 | 0.30 | 0.15 | 0.27 | -0.25 | -0.27 |
|  | p-value | 0.20 | 0.12 | 0.09 | 0.19 | 0.00 | 0.00 | **0.00** | 0.01 | 0.01 | 0.22 | 0.02 | 0.05 | 0.02 |
| CTV Max Weight | r | 0.07 | 0.05 | 0.14 | 0.14 | **-0.70** | **-0.83** | **-0.90** | 0.29 | -0.59 | -0.54 | -0.60 | -0.08 | 0.54 |
|  | p-value | 0.55 | 0.67 | 0.24 | 0.23 | **0.00** | **0.00** | **0.00** | 0.02 | 0.00 | 0.00 | 0.00 | 0.52 | 0.00 |
| OAR Max Weight | r | -0.44 | -0.50 | -0.29 | -0.27 | -0.69 | **-0.72** | **-0.80** | -0.53 | -0.62 | -0.47 | -0.63 | 0.50 | 0.53 |
|  | p-value | 0.00 | 0.00 | 0.01 | 0.02 | 0.00 | **0.00** | **0.00** | 0.00 | 0.00 | 0.00 | 0.00 | 0.00 | 0.00 |
| CTV Max Value | r | 0.02 | 0.08 | 0.04 | 0.01 | 0.28 | 0.34 | 0.53 | -0.05 | 0.28 | 0.28 | 0.25 | -0.04 | -0.27 |
|  | p-value | 0.90 | 0.50 | 0.74 | 0.92 | 0.02 | 0.01 | 0.00 | 0.67 | 0.02 | 0.02 | 0.04 | 0.78 | 0.02 |
| OAR Max Value | r | 0.09 | 0.10 | 0.08 | 0.06 | 0.11 | 0.10 | 0.23 | 0.14 | 0.07 | 0.05 | 0.03 | -0.05 | -0.07 |
|  | p-value | 0.44 | 0.43 | 0.50 | 0.60 | 0.36 | 0.47 | 0.06 | 0.28 | 0.59 | 0.66 | 0.80 | 0.71 | 0.59 |
| CTV Volume | r | 0.01 | -0.04 | -0.04 | 0.02 | -0.54 | **-0.73** | **-0.83** | 0.10 | -0.47 | -0.45 | -0.49 | -0.02 | 0.45 |
|  | p-value | 0.92 | 0.75 | 0.76 | 0.90 | 0.00 | **0.00** | **0.00** | 0.40 | 0.00 | 0.00 | 0.00 | 0.90 | 0.00 |
| OAR SP | r | -0.02 | -0.01 | 0.00 | -0.02 | 0.02 | 0.03 | 0.05 | -0.03 | 0.00 | -0.03 | -0.03 | 0.01 | -0.01 |
|  | p-value | 0.84 | 0.94 | 0.98 | 0.89 | 0.87 | 0.80 | 0.68 | 0.78 | 1.00 | 0.84 | 0.79 | 0.94 | 0.93 |
| Normal tissue SP | r | 0.20 | 0.07 | 0.11 | 0.12 | -0.55 | -0.41 | 0.16 | 0.37 | -0.59 | -0.62 | -0.62 | -0.24 | 0.59 |
|  | p-value | 0.10 | 0.58 | 0.36 | 0.31 | 0.00 | 0.00 | 0.17 | 0.00 | 0.00 | 0.00 | 0.00 | 0.06 | 0.00 |
| CTV Density | r | 0.03 | 0.08 | -0.04 | -0.03 | 0.44 | 0.60 | **0.84** | -0.01 | 0.40 | 0.37 | 0.38 | 0.00 | -0.38 |
|  | p-value | 0.81 | 0.53 | 0.75 | 0.81 | 0.00 | 0.00 | **0.00** | 0.91 | 0.00 | 0.00 | 0.00 | 1.00 | 0.00 |
| OAR % on Surface | r | -0.02 | -0.04 | -0.01 | -0.03 | 0.00 | -0.01 | -0.06 | -0.04 | -0.01 | -0.02 | -0.05 | 0.02 | -0.01 |
|  | p-value | 0.84 | 0.76 | 0.93 | 0.83 | 0.98 | 0.95 | 0.61 | 0.75 | 0.94 | 0.89 | 0.69 | 0.86 | 0.94 |
| DTGR | r | 0.08 | 0.15 | 0.13 | 0.08 | 0.07 | 0.05 | 0.00 | 0.17 | 0.05 | 0.05 | 0.02 | -0.14 | -0.04 |
|  | p-value | 0.49 | 0.22 | 0.30 | 0.53 | 0.55 | 0.70 | 0.98 | 0.16 | 0.68 | 0.71 | 0.87 | 0.27 | 0.74 |

Abbreviations: SP = sampling points; r = correlation coefficient. The absolute values of correlation coefficients of 0.7 or more are shown in bold type.
