# Supplementary material for: Analysis of treatment planning time and optimization parameters for inverse planning for intracavitary and interstitial brachytherapy in uterine cervical cancer
Source: J Appl Clin Med Phys. 2025 Jul 14;26(7):e70157. doi: 10.1002/acm2.70157 (PMC12257335; doi:10.1002/acm2.70157)
Supplement: Supplementary file 2 — Supporting Table S2 [file ACM2-26-e70157-s002.docx]

Table S2. Effect of each optimization parameter on the dose-volume indices (multiple regression analysis).

|  |  | Rectum D_2cc_ | Bladder D_2cc_ | Sigmoid D_2cc_ | Small Bowel D_2cc_ | CTV_HR_ 　D_90_ | CTV_HR_ 　D_98_ | CTV_HR_ V_CTV,100_ | Time | V_150_ | V_200_ | DNR | CI | HI |
| --- | --- | --- | --- | --- | --- | --- | --- | --- | --- | --- | --- | --- | --- | --- |
| CTV Min Weight | β | 0.04 | 0.06 | 0.07 | 0.04 | **0.11** | **0.11** | **0.04** | **0.10** | **0.10** | 0.05 | **0.10** | -0.07 | **-0.10** |
|  | p-value | 0.30 | 0.14 | 0.10 | 0.37 | **0.00** | **0.00** | **0.04** | **0.01** | **0.00** | 0.18 | **0.00** | 0.07 | **0.00** |
| CTV Max Weight | β | 0.06 | 0.04 | **0.11** | **0.13** | **-0.26** | **-0.29** | **-0.17** | **0.12** | **-0.39** | **-0.35** | **-0.39** | -0.07 | **0.37** |
|  | p-value | 0.14 | 0.30 | **0.00** | **0.00** | **0.00** | **0.00** | **0.00** | **0.00** | **0.00** | **0.00** | **0.00** | 0.09 | **0.00** |
| OAR Max Weight | β | **-0.21** | **-0.24** | **-0.13** | **-0.08** | **-0.59** | **-0.71** | **-0.86** | **-0.30** | **-0.35** | **-0.18** | **-0.35** | **0.26** | **0.27** |
|  | p-value | **0.00** | **0.00** | **0.00** | **0.05** | **0.00** | **0.00** | **0.00** | **0.00** | **0.00** | **0.00** | **0.00** | **0.00** | **0.00** |
| CTV Max Value | β | 0.00 | 0.02 | 0.01 | 0.00 | **0.06** | 0.04 | 0.01 | 0.00 | 0.07 | 0.07 | 0.06 | -0.01 | -0.06 |
|  | p-value | 0.99 | 0.52 | 0.88 | 0.97 | **0.05** | 0.13 | 0.78 | 0.97 | 0.05 | 0.06 | 0.09 | 0.81 | 0.06 |
| OAR Max Value | β | 0.01 | 0.01 | 0.01 | 0.00 | 0.02 | 0.01 | 0.00 | 0.01 | 0.01 | 0.01 | 0.01 | -0.01 | -0.01 |
|  | p-value | 0.75 | 0.77 | 0.81 | 0.91 | 0.57 | 0.75 | 0.92 | 0.79 | 0.70 | 0.82 | 0.83 | 0.81 | 0.69 |
| CTV Volume | β | 0.01 | -0.01 | 0.00 | 0.00 | **-0.14** | **-0.14** | **-0.04** | 0.02 | **-0.16** | **-0.16** | **-0.16** | -0.01 | **0.16** |
|  | p-value | 0.78 | 0.81 | 0.94 | 0.95 | **0.00** | **0.00** | **0.03** | 0.66 | **0.00** | **0.00** | **0.00** | 0.78 | **0.00** |
| OAR SP | β | 0.01 | 0.00 | 0.01 | 0.00 | 0.01 | 0.01 | 0.00 | 0.01 | 0.01 | 0.00 | 0.00 | -0.01 | -0.01 |
|  | p-value | 0.90 | 0.95 | 0.89 | 0.98 | 0.82 | 0.62 | 0.90 | 0.84 | 0.85 | 1.00 | 0.97 | 0.83 | 0.88 |
| Normal tissue SP | β | 0.07 | 0.00 | 0.03 | 0.02 | **-0.12** | **-0.06** | 0.00 | **0.08** | **-0.14** | **-0.17** | **-0.14** | -0.07 | **0.14** |
|  | p-value | 0.09 | 0.94 | 0.48 | 0.67 | **0.00** | **0.03** | 0.94 | **0.03** | **0.00** | **0.00** | **0.00** | 0.10 | **0.00** |
| CTV Density | β | 0.01 | 0.02 | -0.01 | -0.01 | 0.05 | 0.05 | 0.01 | 0.01 | 0.05 | 0.05 | 0.05 | -0.01 | -0.05 |
|  | p-value | 0.77 | 0.69 | 0.86 | 0.87 | 0.10 | 0.08 | 0.57 | 0.88 | 0.10 | 0.17 | 0.13 | 0.88 | 0.13 |
| OAR % on Surface | β | 0.00 | -0.01 | 0.00 | 0.00 | 0.00 | 0.00 | 0.00 | 0.00 | 0.00 | 0.00 | -0.01 | 0.00 | 0.00 |
|  | p-value | 0.97 | 0.88 | 0.99 | 0.98 | 0.96 | 1.00 | 0.99 | 0.96 | 0.99 | 0.94 | 0.79 | 0.96 | 0.98 |
| DTGR | β | 0.02 | 0.02 | 0.03 | 0.01 | 0.02 | 0.01 | 0.00 | 0.02 | 0.01 | 0.01 | 0.00 | -0.03 | -0.01 |
|  | p-value | 0.61 | 0.58 | 0.45 | 0.85 | 0.60 | 0.77 | 0.99 | 0.52 | 0.72 | 0.71 | 0.94 | 0.45 | 0.77 |

Abbreviations: SP = sampling points; β = standardized coefficient. Regression coefficients were standardized to examine the degree of influence due to the different units and scales of the dose-volume indices. The p-value <0.05 are shown in bold type.
